# Frontal augmentation as an adjunct to orthognathic or facial contouring surgery

**DOI:** 10.1186/s40902-016-0084-y

**Published:** 2016-10-05

**Authors:** Young-Wook Park

**Affiliations:** Department of Oral and Maxillofacial Surgery, College of Dentistry, Gangneung-Wonju National University, 7 Jukheon-gil, Gangneung, 210-702 South Korea

**Keywords:** Esthetic frontal augmentation, Silicone implant, Dentofacial deformity

## Abstract

**Background:**

The dimensions and shape of the forehead determine the esthetics of the upper third of the face. Korean young people consider a broad and smooth, rounded forehead more attractive. As a result, frontal augmentation becomes more popular in patients with dentofacial deformities. Various surgical procedures and materials have been used in frontal augmentation surgery, with associated advantages and disadvantages. Silicone is a good candidate for frontal augmentation. The author presents two cases of esthetic frontal augmentation with a prefabricated silicone implant in female patients with dentofacial deformities.

**Case presentation:**

In case 1, a 24-year-old female patient underwent frontal augmentation surgery with simultaneous maxillomandibular and zygomatic osteotomies to correct facial asymmetry. A silicone implant was fabricated preoperatively using a positive template stone mold of her forehead. In case 2, a 23-year-old female patient underwent total facial contouring surgery including frontal augmentation for improved facial esthetics. A computed tomography (CT)-guided rapid prototype (RP) model was used to make the silicone implants. The operative procedure was safe and simple, and the silicone implants were reliable for a larger degree of frontal augmentation. Six months later, both patients had recovered from the surgery and were satisfied with their frontal shape and projection.

**Conclusions:**

Frontal augmentation with silicone implants can be an effective adjuvant strategy to improve facial esthetics in patients with a flat and narrow forehead who undergo orthognathic reconstruction or total facial contouring surgery.

## Background

The goal of orthognathic surgery is to achieve a more balanced and harmonious facial appearance as well as a functional stomatognathic system. Oral and maxillofacial surgeons usually improve the appearance of the middle and lower third of the face by using orthognathic osteotomies and conventional facial contouring surgery such as malarplasty, mandibular angle surgery, and genioplasty, and sometimes with the use of alloplastic materials [1]. However, some patients have a deficient forward projection of frontal bone, which is considered unattractive and even ugly. In general, the female forehead has no supraorbital bossing and is basically a continuous curve, whereas supraorbital bossing and flatness above the bossing is a characteristic feature of the male forehead [2, 3]. Thus, frontal augmentation surgery is required to improve the facial esthetics in female patients with a flat and narrow forehead.

Various surgical procedures have been reported for frontal augmentation. Autologous fat grafting and alloplastic implants such as silicone, expanded polytetrafluoroethylene (ePTFE), methyl methacrylate, hydroxyapatite, and the use of hyaluronic acid with and without radiofrequency have been described, with their associated advantages and disadvantages [4–8]. Autologous fat is the most biocompatible source for frontal augmentation, but the rate of resorption is not predictable. Moreover, the consistency of the forehead is somewhat incongruent with autologous fat, which is too supple. Furthermore, periorbital lipogranuloma after autologous fat injection for cosmetic forehead augmentation has been reported [9, 10].

Silicone implants are not biocompatible due to the higher risk of capsule formation by surrounding tissues [11], sometimes resulting in implant movement after surgery. Despite the disadvantages, the greater degree of augmentation, ease of prefabrication, and long-term maintenance of harmonious shape and consistency lead surgeons to use silicone implants. The author reports two cases of esthetic frontal augmentation with silicone implants in female patients with the chief complaint of dentofacial deformities.

## Case presentation

### Case 1

A 24-year-old female was referred to the Department of Oral and Maxillofacial Surgery from the Department of Orthodontics, Gangneung-Wonju National University Dental Hospital. She was diagnosed with facial asymmetry, and preoperative orthodontic treatment was performed. The orthodontist wanted to correct the maxillary canting and prognathic mandible. However, she had a brachy-type facial shape with a prominent zygoma as well as a narrow and flat forehead, which appeared unattractive. Therefore, the final surgical plan was to perform maxillomandibular orthognathic surgery with mandibular angle reduction, reduction malarplasty, and frontal augmentation to achieve a more balanced face.

**Fig. 1:**
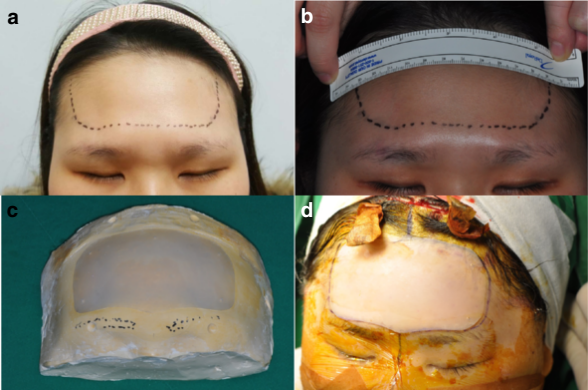
Outline of silicone implant was marked (**a**) and measured (**b**) clinically. We checked the adaptability of prefabricated silicone implant in stone model (**c**) and finally in operation (**d**)

Preoperatively, we fabricated a custom-made silicone implant. First, a positive template stone mold of the patient’s forehead was created from the negative template of the forehead via alginate impression. Next, the area of the forehead that required augmentation was outlined chairside with the patient. Both lateral borders were the medial borders of the temporalis muscle, the inferior border was the supraorbital ridge, and the superior border was about 2.5 cm above the hairline. In this step, the patient’s clinical information should be considered. Clenching was helpful to identify the border of the temporalis muscle. The supraorbital ridge was also palpated carefully to determine the inferior border on the patient’s forehead, which was transferred to the stone mold. The superior border was also determined by using the patient’s hairline to hide the border of the silicone implant (Fig. 1a, b).

After the completion of the design of the implant, fabrication was performed by JMmedi (Seoul, Republic of Korea). We verified the correct shape and position of the silicone implant chairside with the patient. The gross contour and borders of the implant were rechecked on the patient’s face. After confirmation, the implant was sterilized with ethylene oxide gas in preparation for surgical implantation. The thickness of the implant was 4.5 mm at the center (Fig. 1c, d).

On Dec 22, 2015, under general anesthesia with nasotracheal intubation, surgical field preparation and draping were performed in the usual manner. To correct the facial asymmetry, a maxillary LeFort I osteotomy and mandibular sagittal splitting ramus osteotomy (SSRO) were performed. During the SSRO, mandibular angle ostectomy was performed to narrow the lower third of the face. Next, reduction malarplasty was performed to decrease the midfacial width.

**Fig. 2:**
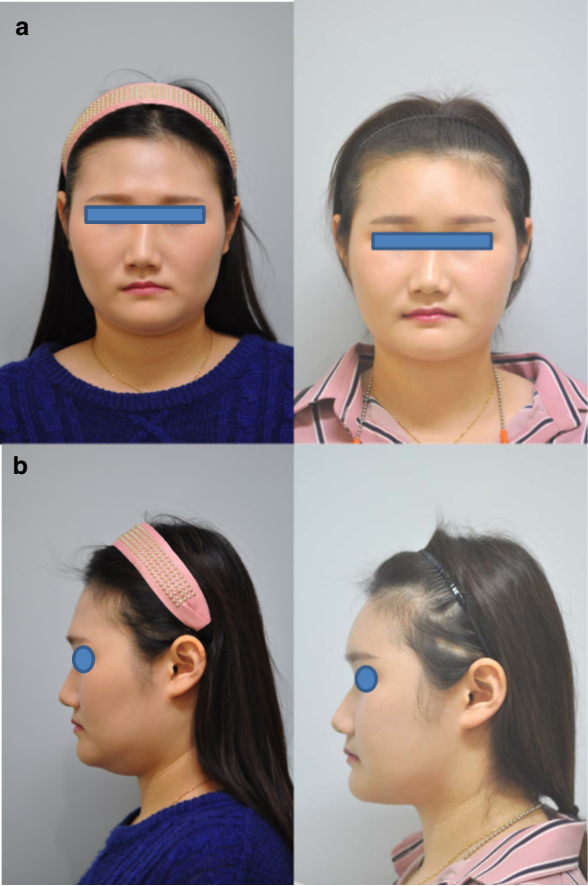
Preoperative and postoperative frontal (**a**) and lateral (**b**) photograms in case 1, 6 months after surgery

After removal of the nasotracheal anesthetic tube, orotracheal intubation and redraping were performed. The entire forehead area was injected with 30 ml of tumescent solution (normal saline 1 L, 8.4 % sodium hydrogen carbonate solution 10 ml, 2 % lidocaine 40 ml, 0.1 % epinephrine 1 ml). After a wait of 20 min, a 4-cm incision down through the periosteum was made with a no. 10 scalpel in the mid-scalp area, about 3 cm superior to the hairline. Subperiosteal dissection was performed to expose the supraorbital rim, carefully avoiding damage to the supraorbital neurovascular bundles. The dissection was fully extended so that the silicone implant could freely rest in the intended position without buckling or being prevented from proper positioning. After proper positioning, the implant was inserted. After profuse irrigation with antibiotic solution, the scalp incision was approximated with surgical staples without a drain. The patient safely recovered from maxillomandibular orthognathic and facial contouring surgery. The 7-month follow-up photographs are presented in Fig. 2.

### Case 2

A 23-year-old female desired esthetic facial contouring surgery, with the complaint of a long and flat face. Unfortunately, at the time of initial diagnosis, she had already undergone orthodontic treatment at a local clinic, not preoperative surgical orthodontics, for protruding lips. Therefore, maxillomandibular orthognathic osteotomies were precluded. Through photographic and cephalometric analysis, and repeated interviews with the patient, esthetic frontal augmentation as well as malarplasty and genioplasty was planned.

A custom-made silicone implant for frontal augmentation was fabricated by using a cone beam CT (CBCT)-based three-dimensional RP model (Ceptech, Seoul, Republic of Korea). The thickness of the implant was 4 mm at the center. Several holes were created in a uniform distribution across the implant to facilitate tissue ingrowth and add implant stability. After carving for adjustments, the silicone implant was sterilized with ethylene oxide gas.

On Jan 13, 2016, under general anesthesia with nasotracheal intubation, narrowing genioplasty and reduction and rotational malarplasty were performed. Then, the anesthetic tube was changed from the nasotracheal to the orotracheal position. After redraping, frontal augmentation was performed as described above. We marked the center of the silicone implant as a V-shaped wedge to ensure proper positioning after insertion. The implant was rolled for ease of insertion because it was larger than the length of the coronal incision. After insertion, it was unrolled with a proper instrument. The implant was carefully palpated through the skin layer to ensure proper positioning. The coronal incision was approximated with surgical staples. A pressure dressing was applied on the forehead, and middle and lower face, using an elastic bandage and plaster.

**Fig. 3:**
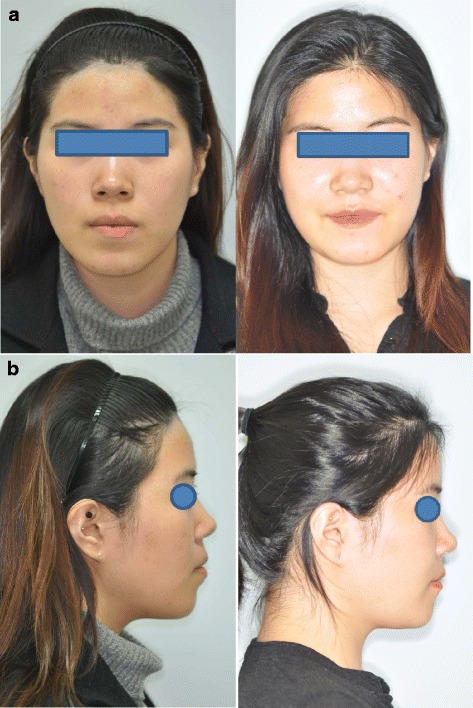
Preoperative and postoperative frontal (**a**) and lateral (**b**) photograms in case 2, 6 months after surgery

Two weeks later, we surgically removed a hematoma around the coronal incision area to dissolve ongoing swelling with fluctuance. After surgical intervention, additional antibiotics and nonsteroidal anti-inflammatory drugs were prescribed. When the postoperative swelling had subsided, the patient complained of a feeling of asymmetry of her forehead. We maintained the implant, and she was satisfied with the esthetic results 6 months later (Fig. 3).

### Discussion

The frontal area that occupies the upper third of the face is one of the critical determinants of facial esthetics, and the demand for frontal contouring surgery is increasing in South Korea. Korean young women consider a broad and convex forehead to appear feminine and more attractive. Some patients who have occlusal disharmony due to a related skeletal deformity also present deficiency in frontal contour and projection. Most of these patients have a horizontally narrow and vertically short forehead. Our patients were also dissatisfied with the dimension and projection of their foreheads. By using a prefabricated silicone implant, we could improve their frontal projection, which resulted in a wider-appearing forehead.

In the use of silicone as a frontal implant, the thickness and dimensions of the implant are important. The silicone implant should be designed with a thicker central region that tapers laterally and smoothly in the temporal area. If the lateral border extends past the border of the temporalis muscle, the silicone implant will be movable after insertion, which results in delayed hematoma formation. The implant should also taper inferiorly toward the superior aspect of the supraorbital ridge. The shape of the implant was a crescent on profile view, with the thickest section in the mid-forehead, which permitted a smooth transition from the augmented area to the periphery. A thickness of 3 to 4 mm is typically necessary to accomplish this task, and no greater than a 6-mm-thick implant has been clinically necessary. In case 1, we prefabricated a 4.5-mm-thick implant because she had mild frontal bossing, and in case 2, a 4-mm-thick implant was used.

Therefore, fabrication of a well-adapted silicone implant is important to decrease the incidence of postoperative complications. To make a well-adapted implant, we first recommend a CT-guided procedure, because a CT-guided RP model reproduces bony contours, enabling fabrication of a more accurate implant. Second, as the hairline is not detected in the RP model, the patient’s clinical information should be considered to determine the superior border of the implant. Third, both lateral borders were also based on the patient’s clinical information.

In case 1, we fabricated the implant from a soft tissue template of the patient’s forehead, and no complications occurred. However, in case 2, an immediate postoperative hematoma developed due to some flaws in surgery, rather than from the adaptability of the silicone implant itself. Comparing to case 1, the adaptability of silicone implant to the exposed frontal bone was better, which could be clinically checked by the movability of the implant when the operator pulled up the surrounding soft tissues. The author thought that the hematoma was induced by the extended scalp incision and inadequate hemostasis, as well as insufficient seating of the silicone implant. The implant appeared to be slightly out of the planned position, but buckling was not perceived. Therefore, the author maintained the implant, which was ultimately esthetically acceptable to the patient. Postoperatively, there was no complaint of foreign body sensation or discomfort related to the implant. However, a rare complication of delayed hematoma after forehead augmentation with a silicone implant has been reported [12]. Long-term follow-up will be needed.

## Conclusions

The author presents the clinical outcomes of esthetic frontal augmentation with silicone implants in patients with dentofacial deformities. The operative procedure was safe and simple. Furthermore, the silicone implant was reliable for a larger degree of frontal augmentation. Therefore, silicone augmentation of the forehead can be a valuable adjuvant procedure in orthognathic reconstruction or total facial contouring surgery.
